# AI software as a third reader in breast cancer screening—a prospective diagnostic observational study

**DOI:** 10.1007/s00330-026-12359-0

**Published:** 2026-02-05

**Authors:** Thomas Lehnen, Doris Polenske, Barbara Daria Wichtmann, Nils Christian Lehnen

**Affiliations:** 1 MVZ KERN Radiologie Lehnen/Polenske, Gelsenkirchen-Buer, Germany; 2 Clinic for Neuroradiology, University of Bonn, University Hospital Bonn, Bonn, Germany; 3 German Center for Neurodegenerative Diseases, Bonn, Germany

**Keywords:** Mammography screening, Computer-assisted diagnosis, Double reading

## Abstract

**Objective:**

Despite advances in mammography screening, some cancers remain undetected, prompting the evaluation of artificial intelligence (AI) as an independent third reader to reduce missed cancers.

**Materials and methods:**

In this prospective study, women eligible for the German Mammography Screening were enrolled at six sites belonging to one screening unit between August 2023 and February 2024. Each mammogram underwent double reading and was independently analyzed using Transpara, an AI-based detection software. Cases rated BI-RADS 4 or 5 by any reader or given a risk score of 10 by the software were reviewed in a consensus conference. Endpoints included: primary—cancer detection rate (CDR) and positive predictive values (PPV); secondary—analysis of cancers detected only by the software or missed by it.

**Results:**

15,356 female participants (mean age 58.6 ± 5.6 years) were included. Overall, 115 breast cancers were detected (CDR triple reading: 0.75%; 95% CI: 0.62%, 0.90%). CDR of double reading and standalone AI was 0.68% (95% CI: 0.56, 0.83%) and 0.66% (95% CI: 0.54, 0.81%). Using Transpara as a third reader increased the detection rate by 9.5% (95% CI: 4.7%, 16.8%) compared to double reading (*p* = 0.002). The PPV for consensus-conference referrals was 5.1% (95% CI: 4.2%, 6.1%), lower than double reading 7.5%(95% CI: 6.2%, 9.0%; *p* < 0.001). For recalled cases, the PPV was 13.7%(95% CI: 11.5%, 16.2%) versus 15.2% (95% CI: 12.6%, 18.1%; *p* < 0.001). All nine invasive cancers detected solely by AI were Luminal-A-like cancers. Among 13 cancers missed by the software, four were triple-negative.

**Conclusion:**

Adding Transpara as an independent third reader improved detection rates, mainly by identifying additional Luminal-A-like cancers, and increased the workload to the consensus conference and the number of recalled cases.

**Key Points:**

***Question*** *Does the integration of AI software as an independent third reader improve cancer detection rates in mammography screening without increasing false-positive findings and recall rates?*

***Findings*** *AI as an independent third reader increased cancer detection by 9.5%, mainly identifying Luminal-A-like cancers, significantly decreasing the positive predictive values of cases referred to at the consensus conference and increasing the number of recalled cases.*

***Clinical relevance*** *Using AI as an independent third reader enhances mammographic cancer detection by offering radiologists complementary sensitivity, especially for low-risk lesions. However, maintaining human readers is essential, as AI may miss aggressive subtypes like triple-negative breast cancers.*

**Graphical Abstract:**

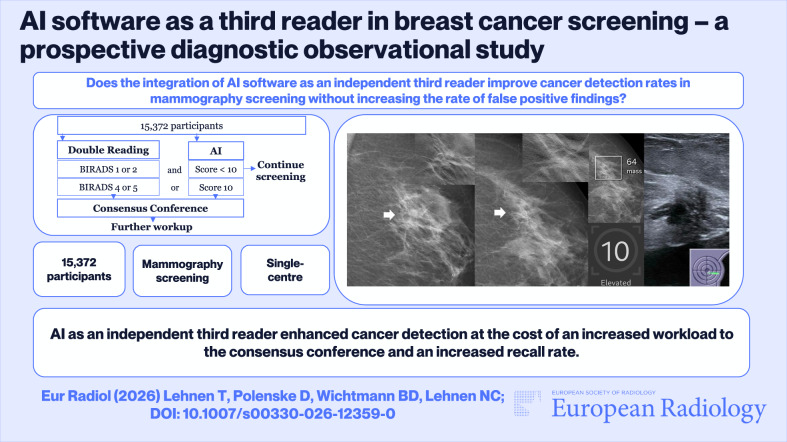

## Introduction

Breast cancer remains a leading cause of cancer-related morbidity and mortality in women [1, 2]. Mammography screening has reduced breast cancer mortality in many countries [3, 4]. Nevertheless, not all cancers are detected by screening: data from the German mammography program (2011–2016) reported detection rates of 69.9–71.7% [5], despite double reading to improve sensitivity while maintaining specificity. Transpara (ScreenPoint Medical), a convolutional neural network–based, FDA-approved and CE-marked AI software, correctly classifies most screen-detected and about half of interval cancers [6, 7]. In the Mammography Screening with Artificial Intelligence (MASAI) trial, the first randomized controlled trial implementing AI software to a mammography screening program, the software improved the cancer detection rate and reduced radiologists’ workload, while maintaining stable recall and false-positive rates [8]. In MASAI, the software triaged exams to single reading (low/intermediate risk) or double reading (high risk) and provided decision support to radiologists. Other prospective studies evaluated AI as an independent third reader. In ScreenTrustCAD, Dembrower et al assessed double reading plus AI as an independent third reader, with radiologists blinded to AI results. Examinations exceeding a predefined risk score entered the consensus conference even if not selected by radiologists. They found triple reading to be superior to conventional double reading in terms of cancer detection [9]. Ng et al prospectively implemented an AI software as an additional reader to standard double reading. In this AI-assisted workflow, cases that were classified as ‘no recall’ by both radiologists but flagged by the AI system were not discussed at the consensus conference; instead, they were reviewed separately by a single radiologist for potential recall. They found a 7% relative increase in cancer detection rate compared to standard double reading [10].

We hypothesized that AI, as an independent third reader, would improve sensitivity without increasing false-positive or recall rates. Additionally, we analyzed sensitivity differences between AI and human readers by tumor characteristics and prognostic subtype.

## Materials and methods

### Ethics approval

This prospective study was approved by the local ethics committee, and informed consent from the study participants was obtained prior to inclusion.

### Study sample

Between August 2023 and February 2024, women eligible for the German Mammography Screening Program were consecutively enrolled at six sites of the authors’ screening unit in this single-center, prospective study. The only inclusion criterion was screening eligibility; participants with incomplete data due to early termination were excluded.

### AI software

Transpara (ScreenPoint Medical) is a convolutional neural network–based software assisting radiologists in detecting breast cancer on mammograms [11]. Detailed training data are proprietary; high-level information has been published. Version 1.7, trained on > 200,000 mammograms including > 10,000 biopsy-proven cancers and prior false negatives, assigns each exam a risk score from 1 to 10 (10 = highest risk). Suspicious regions are highlighted and scored 1–100, with values ≥ 60 yielding a risk score of 10. For this study, version 1.7.3 was used without recalibration. According to the manufacturer, it shares the same algorithm as version 1.7.0 used in the MASAI trial.

### Study design

Two of ten certified screening radiologists independently read each 2D mammogram according to BI-RADS [12]. Examinations rated BI-RADS 4 or 5 by any reader were reviewed in a consensus meeting (two readers + one program director) to determine further work-up. Findings were classified as densities, microcalcifications, or architectural distortions. Spiculated margins or radiating lines without central masses were categorized as distortions. Each mammogram was also analyzed by the AI software, treated as an independent third reader. Cases with a risk score of 10 were added to the consensus meeting. Radiologists were blinded to AI results during primary reading but had access during consensus. Based on additional imaging or clinical results, participants underwent biopsy or routine follow-up after 2 years. A case was positive if cancer was histologically confirmed, and negative if no malignancy was found or no further work-up was recommended.

### Primary endpoint

The primary endpoint was the cancer detection rate (CDR) and the positive predictive value (PPV) for consensus referrals and recalled cases of triple reading with AI as a third reader and conventional double reading.

### Secondary endpoints

Secondary endpoints were (1) sensitivity differences between AI and an average reader for cancers detected only by AI or missed by it, stratified by key tumor characteristics; (2) the effect of replacing one reader with AI on sensitivity.

PPV was defined as the proportion of cancers among consensus or recalled cases. Invasive cancers with positive hormone receptor, negative HER2, and Ki-67 < 20% were classified as Luminal-A-like; all others as non-Luminal-A-like [13].

### Post hoc analysis

**Fig. 1:**
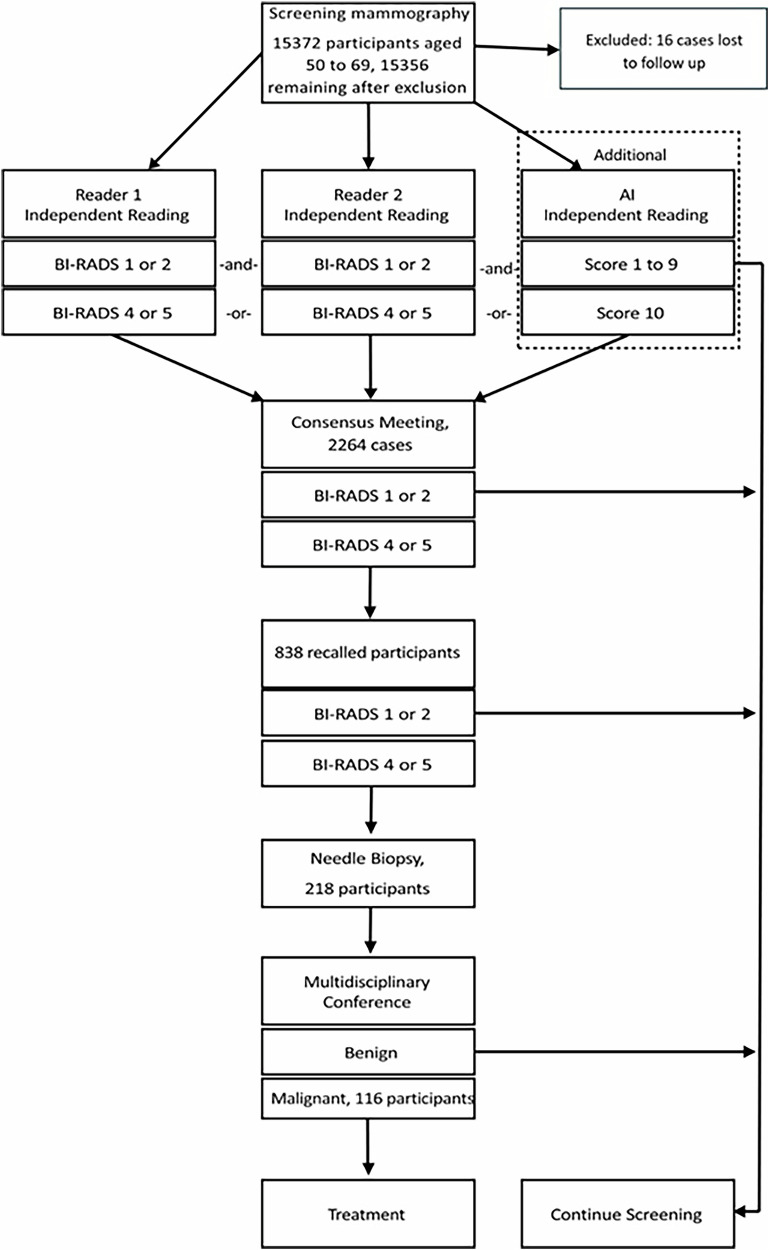
Study design

A post hoc analysis included score 9 densities but excluded score 10 cases based only on calcifications with regional scores < 75 to assess potential gain in detection versus workload, as no carcinomas were found among such score 10 cases. The process of inclusion and exclusion and the study design are summarized in Fig. 1.

### Statistical analysis

Statistical analyses were conducted with R, version 4.4.1, and RStudio, version 2024.04.2 + 764. Confidence intervals for single proportions were calculated using the Clopper-Pearson method. Sensitivity differences between AI and the average reader were analyzed using a paired design. Fractional discordant counts from averaging across readers were rounded to the nearest whole number to match the overall true-positive difference. Ninety-five percent confidence intervals for paired sensitivity differences were calculated with DTComPair [14] using the Agresti–Min method, and group differences were assessed with McNemar’s test or McNemar’s exact test. For PPV analyses, the DTComPair package and the compbdt script were used [15]. For comparing PPV between the average reader-plus-AI and the three-reader strategy, a χ² test was applied. To control the family-wise error rate across the three primary hypotheses, a Bonferroni correction was applied, setting significance at *p* = 0.0167. Secondary and post hoc analyses were exploratory without correction for multiple testing; *p*-values are reported descriptively. ROC analysis was performed using software-provided risk scores and the pROC package [16]. No statistical power analysis was performed for this study.

## Results

### Study sample

Of 15,372 enrolled women, 16 (0.1%) were lost to follow-up and excluded, leaving 15,356 participants. Among them, 115 breast carcinomas (CDR triple reading: 0.75%; 95% CI: 0.62–0.90%) were diagnosed, including 99 invasive cancers. One lung cancer metastasis detected was not included in the final analysis. Baseline, screening, and tumor characteristics are summarized in Table 1.

**Table 1:** Screening results and tumor characteristics

Characteristic	Number	%	95% CI
Participants	15,356	100.0%	
Mean age (years)	58.6 ± 5.6		
BI-RADS 2, 3, B2, B3	15,240	99.24%	(99.1–99.4%)
Breast cancers	115	0.75%	(0.6–0.9%)
Metastasis	1	0.01%	(0.00–0.04%)
Breast cancers, histology confirmed, total	115	100.0%	(96.8–100.0%)
DCIS	16	13.9%	(8.2–21.6%)
Invasive, NST and not lobular	77	67.0%	(57.6–75.4%)
Invasive lobular	14	12.2%	(6.8–19.6%)
Invasive, mixed with lobular component	8	7.0%	(3.1–13.3%)
Invasive, immunohistochemical features, total	99	100.0%	(96.3–100.0%)
ER positive, HER2 negative, Ki-67 < 20%	59	59.6%	(49.3–69.3%)
ER positive, HER2 positive or negative, Ki-67 ≥ 20%	22	22.3%	(14.5–31.7%)
Her2 positive, ER negative	6	6.1%	(2.3–12.7%)
Triple negative	12	12.1%	(6.4–20.2%)
Invasive, grade (Nottingham scale), total	99	100.0%	(96.3–100.0%)
1	25	25.3%	(17.1–35.0%)
2	54	54.6%	(44.2–64.6%)
3	19	19.2%	(12.0–28.3%)
Not documented	1	1.0%	(0.0–5.5%)
Tumor stage TNM classification	115	100.0%	(96.8–100.0%)
is (postoperative)	16	13.9%	(8.2–21.6%)
1a (postoperative)	11	9.6%	(4.9–16.5%)
1b (postoperative)	28	24.3%	(16.8–33.2%)
1c (postoperative or clinical)	30	26.1%	(18.3–35.1%)
2 (postoperative or clinical)	12	10.4%	(5.5–17.5%)
4 (postoperative or clinical)	1	0.9%	(0.0–4.8%)
1 (postoperative after neoadjuvant chemotherapy)	12	10.4%	(5.5–17.5%)
2 or 3 (postoperative after neoadjuvant chemotherapy)	3	2.6%	(0.5–7.4%)
Not documented	2	1.7%	(0.2–6.1%)
Nodal stage TNM classification	99	100.0%	(96.3–100.0%)
N0	82	82.8%	(74.0–89.7%)
N positive	16	16.2%	(9.5–24.9%)
Not documented	1	1.0%	(0.0–5.5%)

*BI-RADS* Breast Imaging Reporting and Data System, *B* pathological grading system, *DCIS* ductal in situ carcinoma, *NST* not specified tumor, *ER* estrogen, *HER2* human epidermal growth factor receptor 2, *is* in situ

### Impact on detection rate and PPVs

The software’s CDR was 0.66% (95% CI: 0.54–0.81%, 102/15356) with an AUC of 0.94 (95% CI: 0.92–0.96). Ten of 115 cancers (8.7%; 95% CI: 4.2–15.4%) were detected solely by the software, increasing the detection rate by 9.5% (95% CI: 4.6–16.8%; *p* = 0.002) versus double reading (CDR double reading: 0.68%; 95% CI: 0.56–0.83%, 105/15356). Nine of these were invasive, and one was ductal carcinoma in situ (DCIS).

**Fig. 2:**
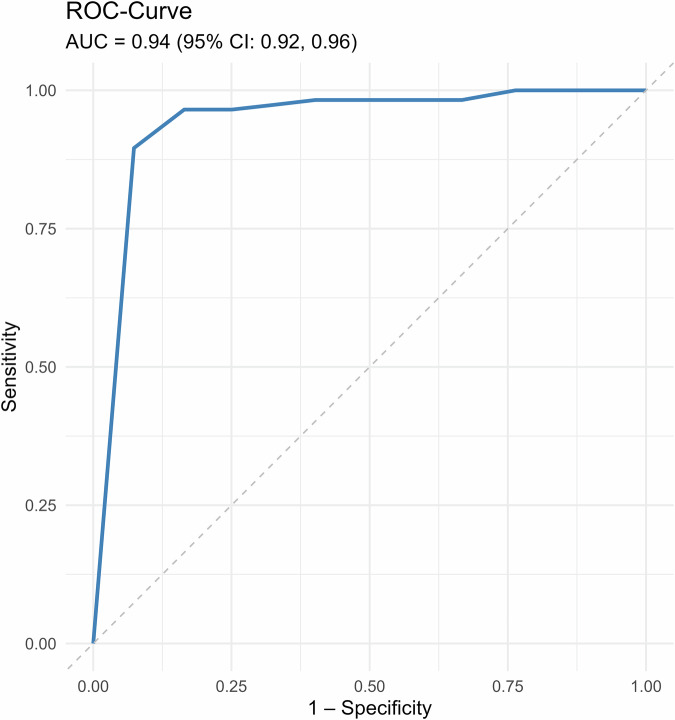
ROC curve of the software as an independent, single reader

PPV for all consensus cases, including those selected by AI, was 5.1% (95% CI: 4.2–6.1%; 115/2264), 2.4 (95% CI: 2.0, 2.8) p.p. lower than with double reading (7.5%; 95% CI: 6.2–9.0%; 105/1403; *p* < 0.001, rPPV = 0.68 [95% CI: 0.64, 0.72]). PPV for recalled cases was 13.7% (95% CI: 11.5–16.2%, 115/838) for triple reading, 1.5 (95% CI: 0.7, 2.3) p.p. lower than the 15.2% for double reading (95% CI: 12.6–18.1%, 105/689; *p* < 0.001, rPPV = 0.9 [95% CI 0.85, 0.95]). Sensitivities and PPVs are summarized in Table 2; the ROC curve is shown in Fig. 2.

**Table 2:** Sensitivities and PPVs of reading instances and reader combinations

	Sensitivity (% (95% CI), *n*/*N*)	PPV before consensus meeting (% (95% CI), *n*/*N*)	PPV recalled cases (% (95% CI), *n*/*N*)
2 readers + AI	100.0 (96.8–100), 115/115***	5.1 (4.2–6.1), 115/2264	13.7 (11.4–16.2), 115/838
2 readers	91.3 (84.6–95.8), 105/115	7.5 (6.2–9.0), 105/1403**	15.2 (12.6–18.1), 105/689**
1 reader + AI	96.5 (91.3–99.0), 111/115	6.0 (4.9–7.1), 110/1848	15.6 (13.0–18.5), 110/704
Reader (average of 10)	76.5 (67.7–83.9), 88/115	9.9 (8.0, 12.1), 88/887	17.8 (14.5–21.4), 88/494
AI	88.7 (81.4–93.8), 102/115	8.3 (6.9–10.0), 102/1223	24.1 (20.1–28.4), 102/424
AI modified*	93.0 (86.8–96.9), 107/115	6.1 (5.0–7.3), 107/1759	(Not applicable)

*PPV* positive predictive value, *AI* artificial intelligence

* Waiving lesions based on calcification findings with region scores < 75 while adding score 9 cases solely based on lesions classified by the AI as density (mass) or density/calcification for being presented in the consensus conference: Minimum values in case no additional cancers would be found

** Significant advantage compared to screening with AI as third reader

*** Significant advantage compared to double reading

### Sensitivity by tumor features: AI-only and AI-missed, compared with average reader

Cancers detected or missed by AI and their characteristics are listed in Table 3; corresponding sensitivities are shown in Table 4.

**Table 3:** Characteristics of cancers solely detected by AI and cancers missed by AI

AI score	ER status	HER2 status	Ki-67 (%)	T stage	G status	Histology (WHO)	Lesion consensus meeting (D, AD, C)	Lesion software (D, C)
10	Positive	-	-	is	2	DCIS	C	C
10	Positive	Negative	8	1c	2	Lobular	D	D
10	Positive	Negative	8	1c	2	Lobular	D, AD	D
10	Positive	Negative	8	1a	1	Mixed	AD	D
10	Positive	Negative	9	1b	2	NST	D, AD	D
10	Positive	Negative	12	1b	2	Lobular	AD	D
10	Positive	Negative	12	1a	1	Lobular	D, AD	D
10	Positive	Negative	13	1b	2	NST	D	D
10	Positive	Negative	15	y2	2	Lobular	AD	D
10	Positive	Negative	16	1c	2	mp	D	D
9	Positive	-	-	is	2	DCIS	C	C
2	Positive	-	-	is	1	DCIS	D	-
9	Positive	Negative	2	1c	1	NST	AD	D
9	Positive	Negative	3	1c	1	Lobular	C, D	C, D
9	Positive	Negative	3	1b	1	Tubular	AD	D
(10)*	Positive	Negative	3	1c	2	Mixed	AD	-
9	Positive	Negative	14	1c	2	NST	D	D
9	Positive	Negative	15	1c	2	NST	D	D
9	Positive	Positive	20	y0	3	NST	D	D
7	Negative	Negative	25	y1b	2	NST	D	-
9	Negative	Negative	28	1a/is	3	NST	C	C
2	Negative	Negative	35	1b	3	NST	D	-
6	Negative	Negative	40	1c	3	Metaplastic	D	-

All cancers in this table were nodal negative; ()* = score based on finding in the right breast, cancer of the left breast

C: microcalcification; y: after neoadjuvant therapy; mixed: with lobular component; is: in situ; mp: micro papillary

*ER* estrogen status, *D* density, mass, *AD* architectural distortion

**Table 4:** Sensitivities of the AI and the average human reader for various tumor characteristics

Cancer characteristics	Sensitivity AI (% (95% CI), *n*/*N*)	Sensitivity reader (% (95% CI), *n*/*N*)	Sensitivity difference (% (95% CI))	Sensitivity difference based on discordant cases, AI vs. average reader				
				*p*-value McNemar	Missed by AI, detected by reader (*n*)	Missed by reader, detected by AI (*n*)	Odds ratio	Advantage
All breast cancers	88.7 (81.4–93.8), 102/115	75.7 (66.8–83.2), 87/115	13.0 (3.9–22.2)	0.01	8.5	23	0.4	AI
Luminal-A-like	89.8 (79.2–96.2), 53/59	67.8 (54.4–79.4), 40/59	22.0 (8.7–35.4)	0.004	3.5	16.5	0.2	AI
Non-Luminal-A-like	87.5 (73.2–95.8), 35/40	90.0 (76.3–97.2), 36/40	2.5 (−10.7–15.7)	0.7	4	3	1.3	AR
Invasive lobular	92.9 (66.1–99.8), 13/14	50.0 (23.0–77.0), 7/14	42.9 (16.2–69.6)	0.03	0	7	0	AI
Triple negative	66.7 (34.9–90.1), 8/12	88.3 (51.6–97.9), 10/12	16.7 (−13.7–47.1)	0.6	3	1	3	AR
T1a + T1b	92.3 (79.1–98.4), 36/39	71.8 (55.1–85.0), 28/39	20.5 (5.8–35.2)	0.04	1.5	9.5	0.1	AI
Distortion	91.7 (77.5–98.2), 33/36	66.7 (49.0–81.4), 24/36	25.0 (7.2–42.8)	0.02	2	11	0.2	AI
Density	82.9 (66.4–93.4), 29/35	80.0 (63.1–91.6), 28/35	28.6 (−19.6–13.9)	1	4.5	5.5	0.8	AI

Luminal-A-like: hormone receptor-positive plus Human Epidermal Growth Factor Receptor 2 negative plus cell proliferation marker (Ki-67) < 20%. Non-Luminal-A-like: all immunohistochemical features different from Luminal-A-like. Distortion: with or without density, without microcalcification; Density: without distortion, without microcalcification. AR: average from 10 human readers. All values were rounded to integer for McNemar tests

All invasive AI-only cancers showed a Luminal-A-like IHC profile. Sensitivity was higher for AI (89.8%; 95% CI: 79.2–96.2%, 53/59) than for the average reader (67.8%; 95% CI: 54.4–79.4%, 40/59; *p* = 0.004).

Four of nine invasive AI-only cancers (44.5%; 95% CI: 13.7–78.8%) showed pure lobular histology. AI sensitivity for lobular cancers (92.9%; 95% CI: 66.1–99.8%, 13/14) exceeded that of the average reader (50.0%; 95% CI: 23.0%; 77.0%, 7/14; *p* = 0.016). Invasive cancers detected only by AI appeared as architectural distortions ± densities, without microcalcifications, in 36.4% (95% CI: 26.9–46.6%, 36/99). For this pattern, AI sensitivity (91.7%; 95% CI: 77.5–98.2%, 33/36) exceeded that of the average reader (66.7%; 95% CI: 49.0–81.4%, 24/36; *p* = 0.02). Five of nine invasive AI-only cancers (55.6%; 95% CI: 21.2–86.3%) were small (pT1a/b). For T1a/b cancers, AI sensitivity (92.3%; 95% CI: 79.1–98.4%, 36/39) exceeded that of the average reader (71.8%; 95% CI: 55.1–85.0%, 28/39; *p* = 0.04).

13 of 115 (11.3%; 95% CI: 6.2–18.6%) cancers had a software score < 10 and were thus false negatives (Table 3). They comprised 2 DCIS and 11 invasive cancers; 5 (45.5%; 95% CI: 16.7–76.6%) were non-Luminal-A-like, including 4 triple-negatives. For these non-Luminal-A-like cancers, sensitivities did not differ between AI (87.5%; 95% CI: 73.2–95.8%) and the average reader (90.0%; 95% CI: 76.3–97.2%; *p* = 0.70). The three triple-negative cancers with scores < 9 showed high proliferation (Ki-67 ≥ 25%) and appeared as pure densities without calcifications or distortions. Among all pure-density cancers, sensitivities were similar for AI (82.9% [95% CI: 66.4%, 93.4%, 29/35]) and the average reader (80.0% [63.1%, 91.6%], 28/35; *p* = 0.75). Three non-Luminal-A-like cancers detected by AI would have been missed by the hypothetical average reader, and four vice versa.

### Impact of replacing one reader with the software

Replacing one radiologist with AI yielded a sensitivity of 96.5% (95% CI: 91.3–99.0%, 111/115) compared with AI as a third reader (*p* = 0.063; Table 2). Consequently, five cancers (4.3%; 95% CI: 1.4%, 9.9%) would have been missed. The sensitivity difference from conventional double reading (91.3%, 95% CI: 84.6–95.8%, 105/115) was not significant (*p* = 0.3). The 10 AI-only cancers would have been found at the expense of 4–5 missed cancers.

PPV before the consensus meeting was 5.1% (95% CI: 4.2–6.1%; 115/2264) with AI as a third reader and 6.0% (95% CI: 4.9–7.1%; 110/1848) with one reader replaced. While screening with AI as a third reader yielded a higher number of positive assessments overall (*n* = 2264 vs. 1848), the number of true positives was similar (115 vs. 110, *p* = 0.21).

After consensus, PPV was 13.7% (95% CI: 11.5–16.2%; 115/838 recalls) with AI as a third reader and would have been 15.6% (95% CI: 13.0–18.5%; 110/704 recalls) with one reader replaced (*p* = 0.32).

### Impact of adjustment of the software’s cutoff for cases referred to the consensus conference

Of seven invasive cancers with a lesion score of 9 (Table 3), six were classified by the software as densities. Only one AI-only cancer was based on microcalcifications (lesion score 77), identified as DCIS. Excluding score-10 cases with pure microcalcifications (lesion score < 75) and including score-9 cases containing densities would have yielded a sensitivity increase of the AI from 88.7% (95% CI: 81.4–93.8%, 102/115) to 93.0% (95% CI: 86.8–96.9%, 107/115) and a pre-consensus PPV for AI cases of 6.1% (95% CI: 5.0–7.3%; 107/1759), assuming no additional cancers would have been found, 2.2 (95% CI: 1.8, 2.7) p.p lower than including all score 10 cases (*p* < 0.001, rPPV = 0.73 [95% CI: 0.68, 0.78]).

**Fig. 3:**
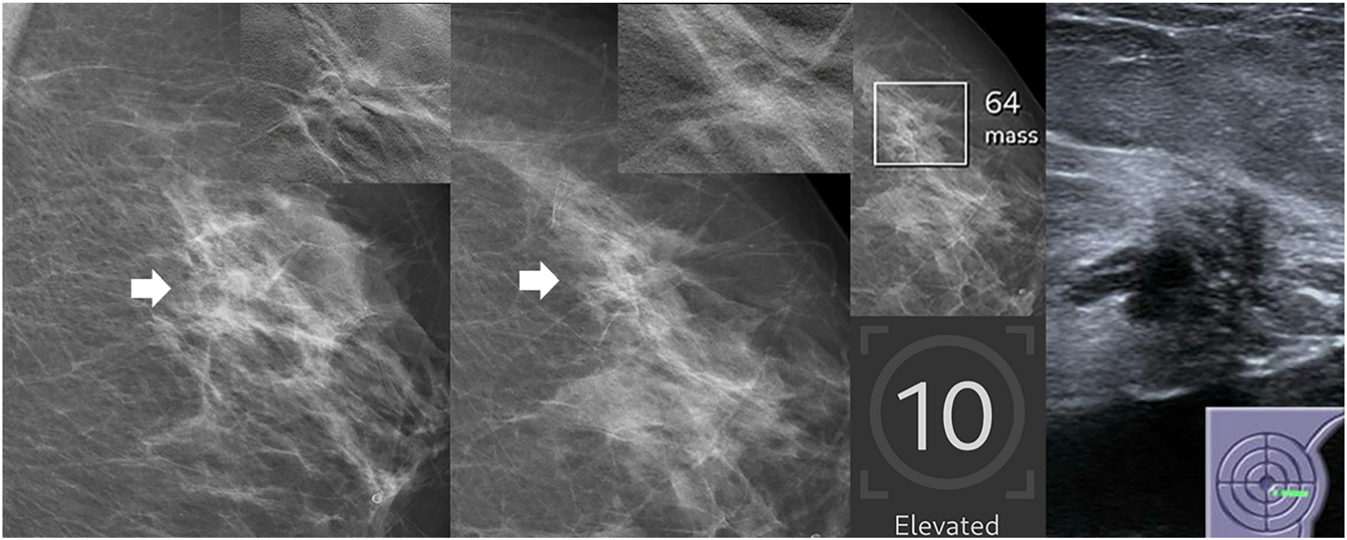
Case solely detected by AI: screening mammogram of a 69-year-old woman with a finding classified by AI as a mass (density), assigned a region score of 64 and a case score of 10. In the consensus conference, the finding was characterized as architectural distortion according to BI-RADS criteria. Histopathology confirmed a multifocal hormone receptor-positive invasive lobular carcinoma with a Ki-67 index of 15%. From left to right: Zoomed-in images of the mediolateral oblique (MLO) and craniocaudal (CC) views of the left breast screening mammogram, centered on the abnormality (arrowheads), with tomosynthesis views from additional imaging in the upper right corners, followed by the corresponding AI analysis and ultrasound correlate

**Fig. 4:**
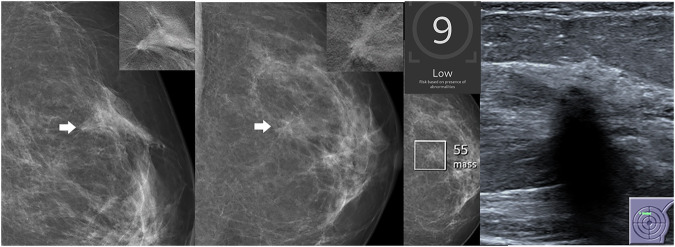
AI-negative case with an AI score just below the cutoff for presentation in the consensus conference. The finding was detected by one of the two human readers. The AI characterized it as a mass (density). During the consensus conference, it was characterized as an architectural distortion. Histopathological analysis confirmed a hormone receptor-positive tubular carcinoma with a Ki-67 index of 3%. Screening mammogram from a 64-year-old woman. From left to right: Zoomed-in images of the mediolateral oblique and craniocaudal views of the screening mammogram of the left breast, centered on the abnormality (arrowheads), with corresponding tomosynthesis views from additional imaging in the upper right corners, followed by the corresponding AI analysis and ultrasound

**Fig. 5:**
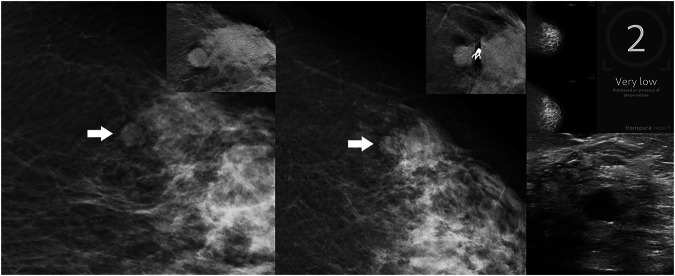
AI-negative finding with a very low case score. The finding was detected by one of the two readers. A 65-year-old woman with a histopathological triple-negative breast cancer with a Ki-67 value of 35%. From left to right: zoomed-in views of the mediolateral oblique and craniocaudal screening mammograms, centered on the abnormality (arrowheads), with corresponding detail views from additional tomosynthesis in the upper right corners, followed by the AI analysis and a magnification view of the ultrasound. Clip marker on the craniocaudal tomosynthesis view

Examples of an AI-only cancer and two false-negative cases are shown in Figs. 3–5.

## Discussion

In this prospective diagnostic study, AI as an independent third reader increased cancer detection compared with double reading, but also raised consensus workload and the number of recalled cases.

The present study included a limited number of pre-specified primary endpoints, for which the family-wise error rate was controlled using Bonferroni adjustment. In contrast, secondary and subgroup analyses were exploratory and not adjusted for multiplicity. Because of substantial subgroup overlap and interdependent diagnostic measures, a global correction would have been overly conservative. Therefore, secondary and post hoc results should be interpreted descriptively and considered hypothesis-generating rather than confirmatory.

The higher detection rate mainly resulted from a high sensitivity of the software for cancers with a Luminal-A-like risk profile according to IHC criteria. In contrast, AI-missed cancers were heterogeneous, including four triple-negative tumors—three with low software scores—all detected by double reading.

AI showed higher sensitivity for lobular histology, small tumors, and spiculations—features frequently associated with Luminal-A-like cancers. The previously reported high AI sensitivity in dense breasts [17] may explain its advantage for Luminal-A-like cancers, as architectural distortions and spiculations—linked to desmoplastic reactions—occur more often in slow-growing than in triple-negative cancers [18–22]. Sensitivity for lobular cancers likely depends on whether they induce architectural distortions. The predominance of Luminal-A-like cancers among AI-only detections may raise concerns of overdiagnosis of indolent tumors [23].

Our findings may inform optimal AI use. As detection reflects three independent readings with partly discordant true positives, removing one or more readers may reduce sensitivity. Alternative reading configurations were not evaluated, and any sensitivity–workload trade-off remains resource-dependent and requires prospective evaluation. Because AI gains were mainly driven by Luminal-A-like cancers, omitting a reader may risk missing non-Luminal-A-like, especially triple-negative, tumors. However, these findings of our study are descriptive in nature due to the low number of cases and are not supported by the current literature. In MASAI, omitting one reader for low-risk cases increased detection of both Luminal-A-like and non-Luminal-A-like cancers [8].

Including score-9 densities in consensus could further increase sensitivity and detection of invasive cancers. The additional workload might be offset by excluding cases with pure microcalcifications and lesion scores below 75.

Further improvements may come from context-driven approaches using asymmetries [24] or prior images [25].

A recent multicenter study found a 17.6% increase in detection when AI aided primary reading rather than acting as an independent third reader [26]. Our 9.5% improvement falls within the wide confidence interval (5.5–30%) of that study. In MASAI, the only randomized trial integrating AI into screening, Transpara was used both as a triage tool for single vs. double reading and as a radiologist support system [27]. Per-protocol analysis showed a moderate but significant increase in detection, with unchanged PPV and recall rates. Interestingly, the screen reading workload could be reduced by 44.2% in this setting [8]. Thus, AI triage and support reduced workload without lowering program sensitivity. In our study, Transpara as an independent third reader increased detection, but also consensus workload and the number of recalled cases. Thus, potential gains in cancer detection from using an AI as a third reader must be balanced against added workload and local resource constraints. Nonetheless, software may still serve as a compelling independent third reader: Knowing its results during primary reading can introduce bias [28], especially when low scores repeatedly occur in triple-negative cancers [28]. By contrast, in MASAI, the Transpara outputs were shown to radiologists to create a “beneficial bias,” reducing false positives in AI-low-risk cases and false negatives in AI-high-risk cases. The recall rate in the intervention group was numerically higher than in the control arm without statistical significance [8]. Furthermore, identifying cancers detected only by the software or missed by it enables cross-checking readers and software and supports cutoff fine-tuning, ideally in adequately powered studies. This level of review is not possible in randomized study designs. Finally, using this approach ensured that all participants benefited from the software.

To distinguish low- from high-risk cancers, tumors were classified as Luminal-A-like or non-Luminal-A-like. The 2021 St. Gallen Consensus defines Luminal A-like tumors as ER-positive, HER2-negative cancers with high PR expression and low Ki-67; no universal Ki-67 cutoff was agreed upon [13]. The proliferation marker Ki-67 has been extensively discussed for differentiating low and high-risk luminal cancers [29, 30]. In this study, we used a Ki-67 cutoff of 20%, acknowledging the inherent arbitrariness and inter-laboratory variability of this threshold. Recent research suggests a 22.5% cutoff for distinguishing between high and low-risk luminal cancers [31]. In our study, cancers classified as Luminal-A-like did not exceed a Ki-67 value of 17%. Additionally, cross-referencing with the Nottingham grading system revealed no non-Luminal-A-like cancers with G1 grading and no luminal-A-like cancers with G3 grading.

We acknowledge several limitations. First, although consensus decisions were based on BI-RADS criteria and context-driven case analysis, including prior images, a bias from the software score, known at this stage of screening, cannot be excluded. In simulating different reading scenarios, such as replacing one reader with the software, we averaged individual reader results—an unreal but pragmatic simplification. Although we assumed each reader worked independently of the software results, this assumption may not be fully true. During the consensus conference, all readers became aware of the software results, potentially introducing bias into subsequent readings. Furthermore, constructing a hypothetical average reader, as done here, did not readily permit statistical tests designed for paired data. Consequently, the PPV comparison using χ² statistics does not fully account for the data’s paired nature, which should be considered when interpreting our results. Second, although our study took place within a single screening unit with six mammography units and ten qualified readers, it was not multicenter; thus, findings may not generalize to programs with different intervals, populations, technologies, AI systems, or workflows. Third, follow-up was insufficient to identify interval or next-round cancers, limiting ground truth to index-round, histologically verified cancers and potentially underestimating AI’s incremental value for cancers missed at initial double reading. Fourth, no prior power analysis was performed, leaving the study underpowered for reliable subgroup analyses. Thus, our subgroup findings are descriptive and must be interpreted cautiously. Still, we believe they are informative and may motivate future research with adequate power.

Using the software as an independent third reader maximized detection by leveraging the complementary strengths of human readers and software. Although sensitivity increased with an added AI reading instance, this required more consensus work and recalls and may be justifiable only where resources allow. We observed the higher detection rate mainly from the software’s higher sensitivity for low-grade invasive cancers. In contrast, detecting more high-risk cancers may depend on multiple independent readings that exploit discordances between readers and software. However, this latter finding requires further investigation in multicenter datasets and larger cohorts to enable meaningful subgroup analyses. Establishing meaningful benefit—beyond detection metrics—necessitates extended post-screening follow-up.

## Supplementary information

Supplementary information

## Abbreviations

AI: Artificial intelligence
CDR: Cancer detection rate
PPV: Positive predictive value
IHC: Immunohistochemistry
p.p.: Percentage points
