# Supplementary material for: AI software as a third reader in breast cancer screening—a prospective diagnostic observational study
Source: Eur Radiol. 2026 Feb 5;36(6):4478–88. doi: 10.1007/s00330-026-12359-0 (PMC13212635; doi:10.1007/s00330-026-12359-0)
Supplement: Supplementary file 1 — Supplementary information [file 330_2026_12359_MOESM1_ESM.pdf]

AI Software as a Third Reader in Breast Cancer Screening – a Prospective Diagnostic Observational Study

ELECTRONIC SUPPLEMENTARY MATERIAL

STARD 2015 Checklist

Section & Topic	No.	Item	☐(N/A)
**TITLE OR ABSTRACT**			
	1	Identification as a study of diagnostic accuracy using at least one measure of accuracy (such as sensitivity, specificity, predictive values, or AUC)	☐
**ABSTRACT**			
	2	Structured summary of study design, methods, results, and conclusions (for specific guidance, see STARD for Abstracts)	☐
**INTRODUCTION**			
	3	Scientific & clinical background, including the intended use and clinical role of the index test	☐
	4	Study objectives and hypotheses	☐
**METHODS**			
Study design	5	Whether data collection was planned before the index test and reference standard were performed (prospective study) or after (retrospective study)	☐
Participants	6	Eligibility criteria	☐
	7	On what basis potentially eligible participants were identified (such as symptoms, results from previous tests, inclusion in registry)	☐
	8	Where and when potentially eligible participants were identified (setting, location and dates)	☐
	9	Whether participants formed a consecutive, random or convenience series	☐
Test methods	10a	Index test, in sufficient detail to allow replication	☐
	10b	Reference standard, in sufficient detail to allow replication	☐
	11	Rationale for choosing the reference standard (if alternatives exist)	☐
	12a	Definition of and rationale for test positivity cut-offs or result categories of the index test, distinguishing pre-specified from exploratory	☐
	12b	Definition of and rationale for test positivity cut-offs or result categories of the reference standard, distinguishing pre-specified from exploratory	☐
	13a	Whether clinical information and reference standard results were available to the performers/readers of the index test	☐
	13b	Whether clinical information and index test results were available to the assessors of the reference standard	☐
Analysis	14	Methods for estimating or comparing measures of diagnostic accuracy	☐
	15	How indeterminate index test or reference standard results were handled	☐
	16	How missing data on the index test and reference standard were handled	☐
	17	Any analysis of variability in diagnostic accuracy, distinguishing pre-specified from exploratory	☐
	18	Intended sample size and how it was determined	☐
**RESULTS**			
Participants	19	Flow of participants, using a diagram. Include the figure number	☐

		(preferably figure 1) or page number	
	20	Baseline demographic and clinical characteristics of participants	☐
	21a	Distribution of severity of disease in those with the target condition	☐
	21b	Distribution of alternative diagnoses in those without the target condition	☐
	22	Time interval and any clinical interventions between index test and reference standard	☐
Test results	23	Cross tabulation of the index test results (or their distribution) by the results of the reference standard	☐
	24	Estimates of diagnostic accuracy and their precision (such as 95% confidence intervals)	☐
	25	Any adverse events from performing the index test or the reference standard	☐
**DISCUSSION**			
	26	Study limitations, including sources of potential bias, statistical uncertainty, and generalizability	☐
	27	Implications for practice, including the intended use and clinical role of the index test	☐
**OTHER INFORMATION**			
	28	Registration number and name of registry	Not applicable
	29	Where the full study protocol can be accessed	Not applicable
	30	Sources of funding and other support; role of funders	Not applicable

*N/A stands for **not applicable** and may be a reasonable choice depending on the type of study performed

RSNA®
